# Adaptive Response to Gillnets Bycatch in a North Sardinia Mediterranean Shag (*Gulosus aristotelis desmarestii*) Population

**DOI:** 10.3390/ani13132142

**Published:** 2023-06-29

**Authors:** Valentina Satta, Angela Pira, Santino Cherchi, Sergio Nissardi, Andrea Rotta, Monica Pirastru, Paolo Mereu, Marco Zedda, Luisa Bogliolo, Salvatore Naitana, Giovanni Giuseppe Leoni

**Affiliations:** 1 Department of Veterinary Medicine, University of Sassari, Via Vienna 2, 07100 Sassari, Italy; valentinasatta80@gmail.com (V.S.); santinocherchi@yahoo.it (S.C.); rotta.andrea73@gmail.com (A.R.); mzedda@uniss.it (M.Z.); luis@uniss.it (L.B.); snaitana@uniss.it (S.N.); 2 Acquario di Cala Gonone, Via La Favorita, 08022 Cala Gonone, Italy; ange.pi@gmail.com; 3 Anthus snc, Via Luigi Canepa 22, 09129 Cagliari, Italy; nissardi@hotmail.com; 4 Department of Biomedical Sciences, University of Sassari, Viale San Pietro 43/B, 07100 Sassari, Italy; pirastru@uniss.it (M.P.); pmereu@uniss.it (P.M.)

**Keywords:** adaptive sex allocation, Mediterranean Shag, bycatch, nestlings sex ratio, sex determination

## Abstract

**Simple Summary:**

Bycatch, a direct negative effect of the fishing industry, is one of the top research priorities in the field of seabird ecology and conservation, being the major cause of seabird mortality. The Mediterranean Shag (*Gulosus aristotelis desmarestii*) is a seabird endemic to the Mediterranean and Black Seas and is considered threatened. The most important site for reproduction for the Mediterranean shag is an islet near Corsica and Sardinia. To evaluate the impact of bycatch in the sex ratio population dynamic, we sampled, extracted DNA and sexed Mediterranean Shags found drowned in nets and newborn chicks in the colony of Corcelli islet, northeast Sardinia. A skewed sex ratio in the effective population was caused by the higher rate of male Mediterranean shags bycaught in nets. At the same time, in the Corcelli colony’s offspring, a skewed sex ratio toward the male sex was recorded. According to the Sex Allocation Theory, the skewed sex ratio of the offspring may represent an adaptive arrangement of the sex ratio by the mothers, to restore the sex ratio in the effective population.

**Abstract:**

Mediterranean Shag (*Gulosus aristotelis desmarestii*) is a seabird endemic to the Mediterranean and Black Seas, recently included in the IUCN list of threatened Species. Most of the reproductive colonies are hosted in Sardinia and surrounding islets. Bycatch in fishing nets is one of the most significant threats for this population. Our work aimed to assess alterations in the sex ratio caused by bycatch and to study the adaptive response of the population to a skewed adult sex ratio. The sex ratio of Mediterranean Shags found drowned in the gillnets near the colonies and that of the nestlings of the Corcelli (northeast Sardinia) colony was determined using the sex-linked polymorphism of the gene Chromobox-Helicase-DNA-binding 1. The data of the shags found drowned in gillnets evidenced a high mortality rate (83.3%; *p* < 0.001) and a larger size of males (35% heavier than females, *p* < 0.05) compared to females, supporting the theory that heavier individuals are able to forage at great depths. With 64.8% of the nestlings being male, the sex ratio of nestlings was statistically different from parity (*p* < 0.05). Furthermore, it was related to the brood size. In one- and two-chick broods, 73% and 70% of nestlings, respectively, were males, while in three-chick broods, only 33% were males. Our data identify the higher rate of male shags drowned in gillnets as a factor causing an alteration of the sex ratio in the Mediterranean Shag population. According to the Sex Allocation Theory, an adaptive adjustment of sex made by adult females restores the Mendelian sex ratio in the population.

## 1. Introduction

In the Mediterranean Sea, unsustainable human activities causing pollution, depletion of fish stocks and increased anthropic pressure on coastal habitats have reduced the availability of food and breeding sites for seabirds [1,2]. The dramatic transformations that the coastal environment has undergone in recent decades have contributed to ensuring that small islands play a key-role in the conservation of a conspicuous part of the Mediterranean biodiversity as they allow the reproduction of most of seabird species. Among them, the European Shag, *Gulosus aristotelis*, has recently been included in the IUCN Red List of Threatened Species and listed as Least Concern [3]. The population of its subspecies, the Mediterranean Shag, *Gulosus aristotelis desmarestii*, which nests on cliffs, islands, and islets restricted to the Mediterranean and Black Seas, is estimated to be lower than 10,000 pairs [4]. In particular, the small islands of northeastern Sardinia and southeastern Corsica host most of the colonies of this population [5].

Current knowledge indicates that Sardinia, and its surrounding islet, hold an important position for the reproduction of Mediterranean Shags [6], whose population is estimated at 1000–1800 pairs [7]; this is why it has been chosen as a flagship species of the ‘Bocche di Bonifacio’ International Marine Park.

Mediterranean Shags form scattered colonies that nest in ravines or caves. Nests are built mainly in winter [8]. Eggs are mostly laid between January and April, in clutch sizes ranging from one to four, of which three are most typical and four are extremely rare [8,9]. The feeding activity is mainly carried out on benthic fish in coastal waters, at deep or medium depths [4].

The main threats to this sub-species are disturbance in breeding sites, trapping in fishing gear (bycatch) and habitat loss due to tourism development [10]. Due to their negative interactions with fisheries, many seabird species are of great conservation interest [11]. Therefore, it is crucial to understand how these interactions impact the population dynamic of these species. Bycatch, a direct negative effect of the fishing industry, is one of the most significant threats faced by this subpopulation [12,13]. Indeed, numerous studies worldwide have shown that bycatch of seabirds in fishing gear potentially poses a considerable risk to these populations [11,14]. Inshore gillnet fisheries are also a major cause of mortality, with 400,000 seabirds killed annually, worldwide [15]. It has been evidenced in Corsica [8] and in the Balearic islands [16] that gillnets and creels are responsible for the killing of a significant number of shags. Genovart and coworkers [12], in a study aimed at evaluating the effect of bycatch in different types of fishing gear, stated that bycatch in gillnets is responsible for 9% of juvenile mortality in Mediterranean Shags. However that study was conducted on the Adriatic sea, where the extent of fishing was lower, while in the other Mediterranean Shag populations, the impact may be stronger [12]. Despite this, data on seabird bycatch mortality for marine ecosystems in the European Union are still limited and insufficient to determine seabird bycatch rates and the resulting impact on seabird populations [15,17]. A better understanding of this phenomenon is needed to determine the full impact of bycatch on Mediterranean Shag populations and on the animal communities of the Corcelli island. Indeed, this has been identified as one of the highest-priority research questions in the field of seabird ecology and conservation [11,18].

Bycatch influences not only the number of individuals killed, but also the composition in terms of sex ratio and age class ratio of the affected populations [18,19,20]. This has led us to hypothesize that the deep-diving foraging strategy is developed early in male individuals of this subspecies. In seabirds, sex bias in survival can accumulate with age and time, resulting in increasingly skewed sex ratios. Several theories predict that natural selection should induce parents to adaptively adjust the sex of their offspring based on predictable conditions (reviews in [21,22,23,24]). Adult sex ratio biases can occur for different causes such as group structure, and sex differences in mortality and dispersal [25]. The theory of sex allocation postulates that females invest in the offspring with the highest reproductive return according to their ability to invest [26], and this should be a key factor favoring selection for the mother’s ability to adjust the sex ratio of her offspring [27]. A sex ratio skewed toward the female sex can have dramatic consequences in a colony because the number of males is the limiting factor in mating, thus increasing the risk of population extinction [28,29,30,31]. Furthermore, most seabirds, including the Mediterranean Shag, are monogamous with obligate biparental care; therefore, deviation from a balanced sex ratio can determine a reduction in the effective population size, in the number of breeders and consequently the number of chicks produced in the colony [29,32,33].

Assessing sex ratios has been proposed as an effective method for testing evolutionary hypotheses regarding parental adjustment of sex allocation [21,26,27,34] and changes in the mating system [35,36,37,38]. Furthermore, adult sex ratio biases in birds have been estimated to be significantly more severe in populations of globally threatened species than in non-threatened species. This finding has crucial implications for bird monitoring and conservation [39]. The causes and effects of skewed offspring sex ratios are considered to be of great importance [40,41]. In the present study, we investigate the effects of behavioral and anthropogenic factors, in particular foraging and fishery activities, that may influence the sex ratio asset of a population, focusing on the adaptive response of the colony to the high mortality rate caused by the frequent bycatch events of Mediterranean Shags in fishing nets. In order to evaluate whether the higher male mortality during foraging could influence population dynamics, we studied the sex ratio in nestlings of two reproductive seasons in the Corcelli islet colony. The rationale of this study agrees with “sex allocation theory”: individuals are expected to vary the sex ratio of their offspring in relation to the specific fitness benefits of sons and daughters [21,23,26].

## 2. Materials and Methods

### 2.1. Sample Collection

**Figure 1:**
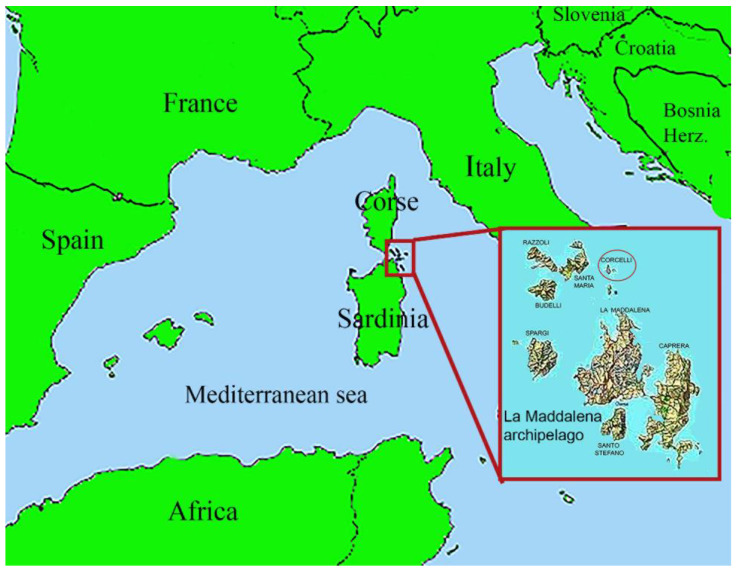
Map of the islands of Corsica and Sardinia in the Mediterranean sea. The red square shows the archipelago of La Maddalena (northeast Sardinia). The island of Corcelli is indicated by a red circle.

The study was conducted on the islet of Corcelli (41°17′45″ N, 9°24′0″ E), in the National Park of the Archipelago of La Maddalena, north-eastern Sardinia (Figure 1), which hosts one of the largest colonies of the Mediterranean Shag.

The habitat is characterized by rocky and sandy bottoms, widely covered with *Posidonia* sea grass beds, ranging from 0 to 70 m in depth. In these waters, artisanal fishing is practiced throughout the year, mainly using gillnets positioned at depths from 10 m to over 50 m (personal observations).

Sample collection was performed over a two-year period. Between 2007–2008, we collected 36 muscle samples from Mediterranean Shags found drowned in gillnets near the Corcelli Island. In addition, 54 nestlings’ feathers were picked up during two ringing campaigns (29 March 2007–30 March 2008) carried out by the National Institute of Wild Fauna, in the Mediterranean Shag colonies of the islet of Corcelli. Broods of the colony, individuated and numbered by consecutive numbers in a metal disk fixed near the brood, were explored for the presence of nestlings. A tail feather was taken from each of the ringed nestlings in each brood, to be used to extract the DNA needed for sex determination. The ring number of nestlings in each brood was registered to determine the sex ratio into broods. Each feather was washed with 70% ethanol, sealed in a marked plastic bag and preserved at low temperatures until their arrival at the laboratory, where it was washed with 70% ethanol and cryoconserved at −20 °C for use in downstream applications.

**Figure 2:**
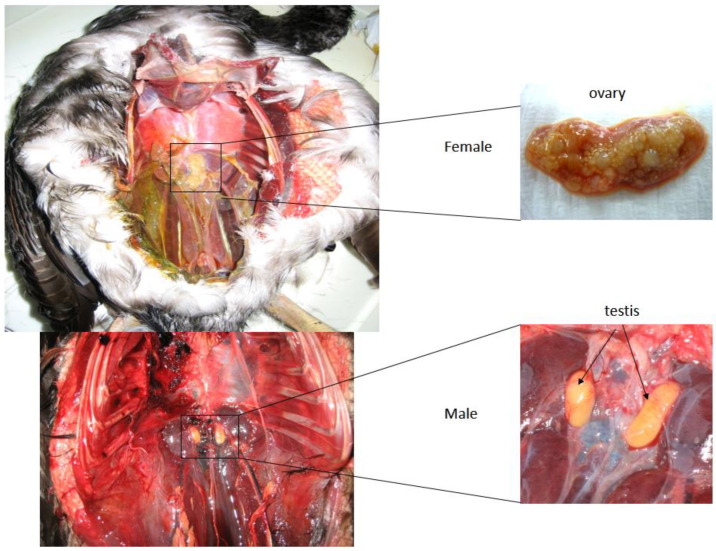
Necropsy of Mediterranean Shag carcasses found dead in gillnets. Samples were classified as male or female according to the morphology of gonads.

The animals found dead in nets were recovered at the moment of net collection by fishers, stored and transported to the laboratory at 4 °C, and immediately classified as adult or juvenile according to plumage, while the morphological evaluation of the gonads after necropsy (Figure 2) allowed the carcasses to be classified as males or females. The stomach was eliminated from carcasses, which were weighed using an analytical scale, and a muscle sample was taken from each specimen of known sex and preserved at −80 °C until molecular analyses.

### 2.2. Sex Determination

We first developed a molecular protocol which allowed us to easily determine the sex of Mediterranean Shags using samples from drowned individuals with known sex determined by necropsy as controls. We sexed the individuals that had drowned in gillnets to monitor any potential impact on the sex ratio of the local population. Additionally, we sexed the colony’s newborn chicks during the same years that we collected the adult samples, to determine whether parental allocation of sex had possibly occurred. A preliminary analysis to define the most effective molecular method for sex determination on this species was performed in samples of adult animals of known sex.

When not expressly specified, reagents come from Merck, Darmstad, Germany.

#### 2.2.1. DNA Extraction

DNA was recovered from the muscle tissue of drowned adult and juvenile Mediterranean Shags using a standard proteinase K lysis technique and phenol and chloroform extraction [42]. Briefly, each sample was incubated under agitation (500 rpm) for at least 1 h at 55 °C in 400 µL lysis buffer solution (10 mM Tris-HCl, pH 8.0, 10 mM NaCl, 1% SDS, 2 mM EDTA) supplemented with 10 µL proteinase K (0.5 mg/mL) and 26 µL of DTT (10 mg/mL). When the tissue was totally dissolved, 400 µL of phenol were added directly to the tube. The solution was stirred, centrifuged at 13,200 rpm for 10 min in a microcentrifuge (Eppendorf microfuge, Eppendorf, Germany), the supernatant was transferred to a clean tube with 200 µL phenol and 200 µL chloroform/isoamyl alcohol 24:1, and centrifuged as before. The aqueous supernatant was transferred in a new clean tube and mixed with 400 µL chloroform/isoamyl alcohol 24:1 and centrifuged as above. The supernatant was mixed with 400 µL isopropanol, stored at −20 °C for 2 h then centrifuged for 15 min at 13,200 rpm. The pellet was washed three times with 70% ethanol before being re-suspended in 100 µL Milli-Q sterile water.

Given the low number of cells in feathers, the DNA extractions were performed by means of a commercial forensic kit (Cst^®^ Forensic DNA Purification Kit, Invitrogen, Waltham, MA, USA), based on positive inducible charge of paramagnetic microbeads and on the DNA affinity for positively charged magnetic microspheres. About 1 cm portions of the feather tip, which contains the most cells, were cut, minced and treated according to the manufacturer protocol. Briefly, each sample was soaked in 1 mL lysis buffer (ChargeSwitch^®^ lysis buffer, Thermo Fisher Scientific, Waltham, MA, USA) with 10 µL of proteinase K, incubated at 55 °C for 1 h, vortexed and briefly spun. The supernatant was added to 200 µL purification buffer containing 20 µL magnetic beads, briefly spun and incubated at room temperature for 1–5 min. DNA, negatively charged by the phosphate groups, bonded to the positive magnetic beads. The sample was placed on a magnet (Magna Rack^TM^, Invitrogen) which attracted the magnetic beads to form a tight brown pellet. The supernatant was aspirated and the pellet was twice washed with 500 µL wash buffer. After the last removal of the wash buffer, the DNA was eluted adding 150 µL TE buffer (10 mM Tris-HCl, 1 mM EDTA, pH 8.5). The nucleic acid concentration was measured using the Qubit^TM^ Fluorometer (Invitrogen).

#### 2.2.2. Sex Assessment

In avian species, the female is the heterogametic sex, having one W and one Z chromosome, whereas males are homogametic (ZZ) [43]. Considering that European shags are slightly dimorphic in size, such that their gender cannot be easily determined from their morphology [44], DNA analysis represents a versatile, effective and non-invasive procedure of discriminating male from female birds.

The method used in this study is based on PCR with primers specific to a highly conserved intron sequence of the Chromobox-Helicase-DNA-binding 1 (CHD1) gene, situated both in the Z and W chromosomes [43]. The length of the non-coding introns usually differs between the CHD1-W and the CHD1-Z genes; therefore, gel electrophoresis immediately reveals one band in the male and two in the female [45,46] in several bird species. To test whether this protocol was effective in discriminating W and Z chromosomes in the Mediterranean Shag, preliminary analyses were performed on samples derived from individuals of known sex. The carcasses of European shags found drowned in nets were autopsied and classified as male or female according to the morphology of reproductive organs, and a piece of muscle from specimens of known sex was used to extract DNA and to test the amplification protocol.

The same protocol was performed to amplify feathers or muscle samples of unknown sex.

#### 2.2.3. Polymerase Chain Reaction

The polymerase chain reaction (PCR) was carried out in a total volume of 15 µL, containing: 30 ng of genomic DNA, 4 µM each of 2550F (5′-GTTACTGATTCGTCTACGAGA-3′) and 2718R (5′-ATTGAAATGATCCAGTGCTTG-3′) primers [43], 1.5 mM of each dNTP, 200 mM Tris-HCl (pH 8.4), 500 mM KCl, 1.5 mM MgCl_2_ and 1 U Taq DNA Polymerase (Invitrogen). PCR amplifications were performed in an MJ research PTC-200 Peltier Thermal Cycler (MJ Research Inc., Deltona, FL, USA) under the following thermal cycling conditions: an initial denaturing step at 95 °C for 2 min followed by 30 cycles, each with denaturing at 95 °C for 30 s, annealing for 1 min at 50 °C, and extension at 72 °C for 1 min. This was followed by a final cycle at 72 °C for 4 min. PCR products were separated by electrophoresis at 90 W on 3% agarose gels stained with ethidium bromide and visualized under UV lights where one band was scored as males and two bands as females.

### 2.3. Statistical Analysis

Comparison of sizes within sex classes (male and female) and within age classes (juvenile and adult) was carried out by Student’s *t*-test (*t*-test). The chi-square test (χ^2^) was used to examine 1:1 deviations in sex ratios in both drowned birds and chicks. The sex ratio of drowned individuals was first assessed, followed by a separate analysis of adults and juveniles. Then, the overall proportion of male nestlings and the sex ratio of annual offspring for 2007 and 2008 were assessed. The data were also analyzed using nests as the unit of analysis in order to evaluate the deviation from parity in the average percentage of males per brood. Finally, clutch size was used as a variable to detect possible changes in sex ratios based on the number of chicks per nest. All statistical analyses were performed using the software Minitab 12 for Windows.

## 3. Results

Of 36 Mediterranean Shags drowned in gillnets, 20 were classified as adults (55%) and 16 as juveniles (45%) based on plumage. Post-mortem inspections highlighted the statistically higher number (χ^2^ = 16, d.f. = 1, *p* = 0.0001) of males (*n* = 30; 83.3%) compared to females (*n* = 6; 16.7%), thus suggesting that males are more active in searching for food at greater depths where the nets are fixed. A significant deviation from the sex ratio parity, with a predominance of males, was found in both juveniles (*n* = 16; 75% males; χ^2^ = 4, d.f. = 1, *p* = 0.046) and adults (*n* = 20; 90% males; χ^2^ = 12.8, d.f. = 1, *p* = 0.0001), without differences between the number of adults and juveniles who became entangled in the nets (χ^2^ = 0.720, d.f. = 1, *p* = 0.396).

As showed in Table 1, in drowned animals, males were found to be significantly heavier than females (males heavier 35% than females; (Mean ± SD: 1.943 ± 0.329 kg vs. 1.416 ± 0.485 kg respectively for males and females, *t*-test: *p* = 0.0335), and non-statistical differences in size were detected between adults and juveniles of the same sex (Mean ± SD: 2.016 ± 0.317 kg vs. 1.877 ± 0.352 kg respectively for adults and juveniles; *t*-test: *p* = 0.271).

**Figure 3:**
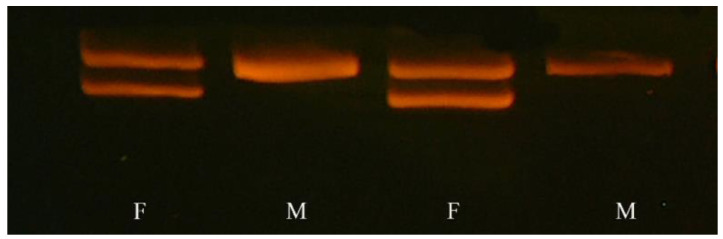
Electrophoresis of CHD1-amplified DNA obtained from muscle samples of Mediterranean Shags drowned in nets. Two bands identified the heterogametic sex (female) and one band the homogametic sex (male).

The PCR products confirmed the sex assigned after the morphological inspection for all 36 individuals used in discriminating male from female specimens (Figure 3). Males, which are the homogametic sex, have shown an electrophoretic pattern characterized by a single band, corresponding to the single sequence located in the Z-chromosome, while in females, heterogametic, two bands can be highlighted, the first being in the Z-chromosome and the second corresponding to the homologous sequence of the W-chromosome. Indeed, in this species, the first intron of the CHD1 gene has a deletion in the Z-chromosome, producing a C-Z-derived PCR product, which migrates more rapidly in the electrophoretic gel than the C-W-derived PCR product, which shows no deletion.

The concordance of the results deriving from electrophoretic and morphological analyses provided confirmation of the effectiveness of the tested molecular methods, which were then applied to infer the sex and the sex ratio of the 54 nestlings in the Corcelli colony. The mean number of nestlings/brood (*n* = 31 brood) was 1.74, with 1.13 males/brood, showing a skewed mean sex ratio/brood, with 64.94% of individuals being males. Among all nestlings in the study population, 64.8% (*n* = 54) were males, statistically different from parity (χ^2^ = 4.741, d.f. = 1, *p* = 0.029).

The sex ratio of annual offspring was significantly shifted towards males in 2007 (*n* = 32, 68.7% males; χ^2^ = 4.5, d.f. = 1, *p* = 0.034), whereas in 2008, when fewer samples were collected from the colony, no significant deviations from parity were found (*n* = 22, 59% males; χ^2^ = 0.727, d.f. = 1, *p* = 0.394; Table 2). However, no statistical difference was found between the sex ratio of 2007 and 2008 nestlings (χ^2^ = 0.533, d.f. = 1, *p* = 0.466) suggesting that the non-significant deviation from parity could be due to the small number of samples collected in 2008 (34.37% lower in 2008 compared to 2007). Furthermore, the sex ratio of nestlings was found to correlate with the clutch size. Considering only the broods of one chick (*n* = 11 broods), it emerged that the sex ratio was strongly shifted towards the male sex, with a total of 73% broods consisting of males and 27% of females (χ^2^ = 2.273, d.f. = 1, *p* = 0.132). A similar result was obtained analyzing data from two-chick broods (*n* = 17 broods), where the mean within-brood sex ratio was 70% males and 30% females (χ^2^ = 5.764, d.f. = 1, *p* = 0.016). On the contrary, considering the three-chick broods (*n* = 3 broods), a preponderance of females was detected, with two nestlings out of three (67%) always females (χ^2^ = 1, d.f. = 1, *p* = 0.317). The sex ratios of one-chick broods and three-chick broods did not statistically differ from parity, probably due to the restricted number of samples, but indicate a trend in the data (Table 2).

## 4. Discussion

This work has allowed us to highlight the alteration of the foraging habitat due to anthropogenic activities and the alteration of the adult sex ratio as environmental and demographic factors that act jointly on a population of Mediterranean Shag, exerting a unidirectional pressure on single parents and determining a non-random sex ratio at birth.

Weighing the carcasses of Mediterranean Shags found drowned in gillnets, we measured 35% greater size in males than in females. There is conflicting information on the influence of size-based mechanisms on sex-related differences in foraging at depths reached by seabirds. Some studies on sexual segregation in foraging have been conducted on size-dimorphic species and a relationship between body size and foraging depth has been highlighted at both an intra- and interspecific level [47,48,49,50,51,52], while other studies have not attributed intra and interspecific differences to body size [47,53,54]. Our results confirm the first hypothesis: also in the Mediterranean Shag, a larger size, as evidenced in the males, is correlated with the ability to forage at greater depths. Since no difference in the size of drowned individuals was found between age classes, it can be inferred that the male-specific deep-diving strategy is a behavioral trait that juveniles acquire early in development and persists in adulthood.

Our data highlighted a sex and age bias in the Mediterranean Shags bycatch in nets near the breeding colonies in La Maddalena archipelago. Similar results were obtained in several seabird species but not in others. In a review reporting the sex composition of seabird bycatch in fisheries worldwide, Dimas and co-workers [55] described that 35% of cases were unbiased, while 46% were male biased and 19% were female biased. The male-shifted sex ratio found in Mediterranean Shags drowned in nets (83% males), suggests the existence of sex segregation in the foraging activity of this species, as described in other seabirds. An important aspect of foraging ecology is the extent to which individuals of different sexes within a population exploit food resources differently [56]. Among seabirds, male and female wandering albatrosses (*Diomedea exulans*) minimize potential competition for food by using distinct fishing areas [57,58], whereas males of both the subantarctic cormorant (*Gulosus albiventer*) and the Japanese cormorant (*Phalacrocorax filamentosus*) carry out much deeper and longer dives than females, thus exploiting different vertical niches in the same fishing area [59]. Our data indicate that male Mediterranean Shags are likely more susceptible than females to death while foraging in nets. This suggest that, in our study area, nets are set at depths at which males catch their prey, while female interaction with fishing gear is limited probably because they forage in shallower, near-shore waters. Assuming that 9% of juveniles of Mediterranean Shags die in the gillnets [12] and from the ratio between adults and juveniles who died in the gillnets obtained in our work (55% vs. 45% of adults and juveniles, respectively), we can deduce that the bycatch in gillnets constitutes 19% of the deaths in the Mediterranean Shag population of the La Maddalena archipelago, and of these, 83.3% are male.

The decline in the male sex ratio in a monogamous population, such as the Mediterranean Shag, attributable to early sex-specific mortality, results in a loss of fitness due to a decrease in the operational sex ratio (OSR), the average ratio of males to females who are ready to mate [60,61]. OSR is a predictor of mate limitation and reproductive success of individuals of a given sex [61] and its alteration can have a strong effect on the mating system through female competition for mates [37]. In this scenario, according to the sex allocation theory, individuals would be expected to vary the sex ratio of their offspring in relation to the specific fitness benefits of sons and daughters [21,25,26]. Our data showed that the mean brood sex ratio was significantly skewed towards males, who accounted for 65% of chicks, with a mean of 1.13 males and 0.61 females per brood. Based on our results, it is conceivable that in the studied population, a process is underway to compensate for the lack of males due to their higher mortality rate in fishing nets when diving for food. At least in diploid organisms, the aggregate of all males in the population has a genetic share in the next generation, which is identical to the genetic share of all females. Therefore, individuals of the minority sex have a higher per capita share, putting a premium on the production of such individuals [62]. Assuming that males, being the under-represented sex in the breeding population, are more valuable than females, we predicted that eggs in a clutch would have a relatively higher chance of producing a male than a female, a situation that was confirmed by our data on the sex ratio within the brood. Females invest in the male sex, which requires more effort than the female, due to the larger size of the males. These results support the existence of a mechanism of optional sex ratio adjustment carried out by adult females. Adaptive adjustment of the sex ratio to maintain high reproductive potential has been found in several bird studies [63,64,65,66]. Indeed, among vertebrates, birds are thought to have unusually direct control of the sex of offspring because the female is heterogametic, and because the sex-determining division in avian meiosis occurs prior to ovulation and fertilization [67].

There have been numerous reports documenting the ability of birds to optionally allocate the sex ratio of their offspring in response to a variety of social and environmental factors [63,64,65,68]. Sex allocation theory predicts a number of situations in which individuals are expected to adjust sex allocation towards male or female production, and there is a large empirical literature supporting these predictions [21,24,69]. The biochemical and physiological mechanisms underlying facultative sex ratio allocation can occur in female birds in the time span from follicle formation to egg laying and most of these mechanisms are related to maternal hormonal profiles (for review, see [70]). Thus, the hormonal state of the female is an important epigenetic mediator between environmental factors experienced by the female and offspring sex adjustment [71]. Hormones circulating in the breeding females during the sex determination period have been shown to influence the sex adjustment [72,73,74]. It has been evidenced in bird species in which females compete for mates that testosterone levels are higher than in females that do not compete for mates [75]. Accordingly, we hypothesize that the lack of male adults and the female-shifted sex ratio of adults might influence the hormonal status during the courtship and mating period, which leads to an adaptive adjustment of offspring sex ratios by mothers. Further studies are needed to investigate the hormonal status of female birds during courtship with or without males.

The molecular sexing method used to determine the sex ratio in Mediterranean Shag was successful for all 90 birds on which it was performed. Furthermore, we have shown that this method is particularly useful when dealing with small quantities of cells from which DNA can be recovered, such as those recoverable from the feather tips. Thus, this technique has been proven to be highly applicable and highly reliable, allowing an un-invasive approach to sample collection, which limits brood handling time, thus reducing the risk of negative repercussion for nestlings. To the best of our knowledge, this is the first time that a reliable and less invasive method for the molecular determination of sex in the Mediterranean Shag has been explored.

## 5. Conclusions

This paper allowed the identification of several ecological and demographic factors operating in concert on the studied Mediterranean Shag population, where they exert a unidirectional pressure on individual parents, thus leading to a non-random, male shifted sex ratio. Specific life history traits of this subspecies, such as the larger size of males and higher variance of their reproductive success, allowed for the accurate prediction of the direction of offspring sex ratio adjustment. However, the higher mortality rates recorded for male individuals seem to represent the main force determining the overcoming of the constraints imposed by the Mendelian segregation of sex chromosomes. This draws attention to the impact of human activities on wild populations and underlines the need to more accurately investigate the effects of ecological and anthropic factors on the genetic variability of this threatened subspecies.

## Figures and Tables

**Table 1:** Summary of the size differences (kg) between male and female individuals bycaught in the nets (*t*-test: a vs. b *p* = 0.0335).

	N	Mean	SD	Minimum	Maximum
F	6	1.416a	0.485	0.860	1.756
M	30	1.943b	0.329	1.620	2.836

**Table 2:** Data on sex ratio of nestlings in the Corcelli population of Mediterranean Shags during the 2007 and 2008 reproductive seasons.

	N Broods	Males	Females	*p*
2007	18	22 (69%)	10 (31%)	0.034
2008	13	13 (59%)	9 (41%)	0.394
Total	31	35 (65%)	19 (35%)	0.029
One-chick broods	11	8 (73%)	3 (27%)	0.132
Two-chick broods	17	24 (70%)	10 (30%)	0.016
Three-chick broods	3	3 (33%)	6 (67%)	0.317

## Data Availability

Not applicable.
